# Prolonged Length of Stay in the Emergency Department and Mortality in Critically Ill Elderly Patients with Infections: A Retrospective Multicenter Study

**DOI:** 10.1155/2021/9952324

**Published:** 2021-07-16

**Authors:** Wonjin Choi, Seon Hee Woo, Dae Hee Kim, June Young Lee, Woon Jeong Lee, Sikyoung Jeong, Kyungman Cha, Chun Song Youn, Sanghyun Park

**Affiliations:** ^1^Department of Emergency Medicine, Incheon St. Mary's Hospital, College of Medicine, The Catholic University of Korea, Seoul, Republic of Korea; ^2^Department of Emergency Medicine, Daejeon St. Mary's Hospital, College of Medicine, The Catholic University of Korea, Seoul, Republic of Korea; ^3^Department of Emergency Medicine, St. Vincent's Hospital, College of Medicine, The Catholic University of Korea, Seoul, Republic of Korea; ^4^Department of Emergency Medicine, Seoul St. Mary Hospital, College of Medicine, The Catholic University of Korea, Seoul, Republic of Korea; ^5^Department of Emergency Medicine, Yeouido St. Mary's Hospital, College of Medicine, The Catholic University of Korea College of Medicine, Seoul, Republic of Korea

## Abstract

**Background:**

This study aimed at investigating whether the length of stay (LOS) in the emergency department (ED) is associated with mortality in elderly patients with infections admitted to the intensive care unit (ICU). Delayed admission to the ICU may be associated with adverse clinical outcomes in elderly patients with infections.

**Methods:**

This was a retrospective study conducted with subjects over 65 years of age admitted to the ICU from 5 EDs. We recorded demographic data, clinical findings, initial laboratory results, and ED LOS. Outcomes were all-cause in-hospital mortality and hospital LOS. A multivariable regression model was applied to identify factors predictive of mortality.

**Results:**

A total of 439 patients admitted to the ICU via the ED were included in this study, 132 (30.1%) of whom died in the hospital. The median (IQR) age was 78 (73, 83) years. In multivariable analysis, a history of malignancy (OR: 3.76; 95% CI: 1.88–7.52; *p* < 0.001), high lactate level (OR: 1.13; 95% CI: 1.01–1.27; *p*=0.039), and ED LOS (OR: 1.01; 95% CI: 1.00–1.02; *p*=0.039) were independent risk factors for all-cause in-hospital admission. Elderly patients with an ED LOS >12 hours had a longer hospital LOS (*p*=0.018), and those with an ED LOS > 24 hours had a longer hospital LOS and higher mortality rate (*p*=0.044,  *p*=0.008).

**Conclusions:**

This study shows that prolonged ED LOS is independently associated with all-cause in-hospital mortality in elderly patients with infections requiring ICU admission. ED LOS should be considered in strategies to prevent adverse outcomes in elderly patients with infections who visit the ED.

## 1. Introduction

The development of medical technology and adoption of healthy lifestyles have led to an increase in life expectancy, which has also resulted in a rise in the elderly population and an aging society [1]. Elderly patients tend to visit the emergency department (ED) more often than the other age groups [2–4]. Indeed, in one study, the proportion of elderly patients with infectious diseases in the ED was significantly higher than that of the other age groups [2]. Additionally, elderly patients have atypical symptoms and signs due to various comorbidities, which makes treatment and diagnosis more difficult and delays admission. Therefore, the probabilities of mortality and intensive care unit (ICU) admission are higher in this population [5–7]. In conclusion, more aggressive diagnostic and treatment protocols are needed for elderly patients than that for other age groups in the ED [7–9], which may lead to a longer ED LOS for elderly individuals with infection.

Specifically, noncommunicable diseases and infection-related conditions such as sepsis are relatively more common in elderly individuals visiting the ED, requiring more specialized and intensive treatment [9]. Other studies have shown that a longer delay in ICU admission in patients with severe disease is associated with more adverse effects on mortality and treatment outcomes [10–12]. However, these studies did not take into account patient age; instead, they evaluated the overall population visiting the ED who were diagnosed with a critical illness and admitted to the ICU. Few studies have investigated the effects of ED length of stay (LOS) on elderly patients.

ED LOS can be prolonged due to diagnostic procedures, therapeutic interventions, consultations with experts in various fields specializing in the treatment of elderly patients, and lack of available beds in the ICU [13]. Elderly patients commonly have multiple underlying diseases at the time of presentation to the ED, and additional diagnostic tests and interventions can lead to an increased ED LOS.

Therefore, this study investigated whether LOS in the ED affects all-cause in-hospital mortality in elderly patients with infectious diseases admitted to the ICU via the ED. In addition, we analyzed whether the hospital LOS of severely ill elderly patients differs based on ED LOS.

## 2. Methods

We performed a retrospective study of patients over 65 years of age who had infectious diseases and were admitted to the ICU via five regional EDs from November 2016 to February 2017 in the Republic of Korea. The inclusion criteria were patients who had a final diagnosis at hospital discharge of pneumonia; infections of the genitourinary tract, gastrointestinal tract, or hepatobiliary system; or other infections. The exclusion criteria were patients who were receiving palliative care and those with missing data. Patients who died within 24 hours of visiting the ED and those who experienced the return of spontaneous circulation after out-of-hospital cardiac arrest were excluded.

Medical records at five regional EDs were reviewed by each institution's trained abstractors. They reviewed the medical records using standardized data collection methods. This study protocol was approved by our institutional review board. As the clinical measurements were routinely collected in the ED and this was a retrospective study, the need to obtain informed patient consent was waived.

We included the following demographic and initial clinical data from the medical ED records: age; sex; comorbidities (hypertension, diabetes, chronic renal disease, cardiovascular disease, and malignancy status); initial vital signs (systolic blood pressure (SBP), diastolic blood pressure, pulse rate, respiratory rate, and body temperature); and Korean Triage and Acuity Scale (KTAS) level in the ED. The KTAS is a triaging tool used in EDs in the Republic of Korea. KTAS Level 1 indicates the most severe condition and Level 5 the least severe condition [14].

The laboratory results (white blood cell (WBC) count, platelet count, and levels of total bilirubin, albumin, and lactate) were investigated in the initial blood test performed in the ED. The Sequential Organ Failure Assessment (SOFA) score was calculated retrospectively based on the information in the medical records. The presence of septic shock in the ED, door-to-antibiotic time (the time in hours from ED arrival to first antibiotic initiation), use of vasopressors and ventilators in the ED, ICU LOS, and hospital LOS were analyzed. The clinical criteria of sepsis and septic shock based on the third international consensus definitions for sepsis and septic shock were defined [15]. ED sepsis care management of each ED was not protocolized, and sepsis management was recommended according to international guidelines based on the Surviving Sepsis Campaign (SSC) [16]. The completion of SSC care bundles within 3 hours was recommended at each ED, which included the following: measure the lactate level, perform blood cultures prior to administration of antibiotics, rapidly administer broad-spectrum antibiotics, and infuse a minimum of 30 mL/kg crystalloid intravenous fluid for hypotension.

ED LOS was defined as the number of hours between arrival at the ED and ICU admission. To analyze the association of ED LOS and all-cause in-hospital mortality, the patients were classified into nonsurvivor and survivor groups based on in-hospital mortality after ICU admission.

The primary outcome of the study was the association between ED LOS and all-cause in-hospital mortality after ICU admission. The secondary outcome was the association between ED LOS and longer hospital LOS.

### 2.1. Data Analysis

All statistical analyses were performed with SPSS ver. 24.0 (SPSS, Inc., Chicago, IL, USA). When investigating predictors of all-cause in-hospital mortality, we explored demographic characteristics, initial vital signs/symptoms, laboratory findings, SOFA scores, and clinical characteristics by comparing survivors and nonsurvivors. The results are expressed as medians with interquartile ranges for continuous variables and as frequencies with percentages for categorical variables. Continuous variables were compared using the Mann–Whitney *U* test. Categorical variables were compared using the chi-square or Fisher's exact test, as appropriate. Logistic regression was used to assess the predictors of all-cause in-hospital mortality. Variables with a *p* value ≤0.05 in univariate analysis were subjected to multivariate logistic regression to identify independent factors of mortality; we calculated odds ratios (ORs) with 95% confidence intervals. A *p* value <0.05 was considered to indicate statistical significance.

## 3. Results

### 3.1. Characteristics of the Study Population

Of the 459 older adults admitted to the ICU via the ED with a suspected infection during the study period, 9 who died within 24 hours of visiting the ED, 4 who experienced the return of spontaneous circulation after out-of-hospital cardiac arrest, and 7 receiving palliative care or who had missing data in the ED were excluded. Thus, 439 patients were included in the study. The most common infection was pneumonia (271 patients; 61.7%) (Table 1).

The median (IQR) age was 78 (73–83) years, and 222 (50.6%) patients were male. Age and gender were not significantly different between the nonsurvivor and survivor groups (*p*=0.996,  *p*=0.376). The most common comorbidity was hypertension (264 patients; 60.1%). However, none of the comorbidity rates except that of malignancy was significantly different between the two groups (29.5% vs. 10.7%, *p* < 0.001). The median systolic BP was lower in nonsurvivors (*p*=0.036). The platelet counts and total bilirubin, albumin, and lactate levels were significantly different between the two groups. Nonsurvivors had a significantly higher median SOFA score (8 (5–11)) than survivors (*p* < 0.001). The door-to-antibiotics time did not show significant differences between the two groups. However, the presence of septic shock, use of ventilation, and use of vasopressors in the ED differed significantly between the two groups (*p* < 0.001, *p* < 0.001, and *p* < 0.001, respectively). The median ED LOS was significantly longer in nonsurvivors (9.0 vs. 8.0 hours, *p*=0.023), whereas the hospital LOS was significantly longer in survivors (13.5 vs. 17.0 days, *p*=0.002) (Table 2).

### 3.2. Prediction of All-Cause In-Hospital Mortality in Elderly Patients with Infections

In the multivariate analysis, the presence of a malignancy, a higher lactate level, and a longer ED LOS were independent risk factors for all-cause in-hospital mortality (OR: 3.76; 95% CI: 1.88–7.52; *p* < 0.001) (OR: 1.13; 95% CI: 1.01–1.27; *p*=0.039) (OR: 1.01; 95% CI: 1.00–1.02; *p*=0.039) (Table 3).

To analyze the association of the outcomes (ICU LOS, hospital LOS, and mortality) with the ED LOS, ED LOS cutoff values of 6, 12, and 24 h were used (Table 4). When an ED LOS cutoff value of 12 h was used, patients with an ED LOS >12 hours had a longer hospital LOS (*p*=0.018). Additionally, when an ED LOS cutoff value of 24 h was used, those with an ED LOS >24 hours had a longer hospital LOS and higher mortality rate (*p*=0.044,  *p*=0.008). However, when an ED LOS cutoff value of 6 h was used, there were no significant differences in the outcomes (Table 4).

## 4. Discussion

In this study, we found ED LOS to be an independent factor influencing all-cause in-hospital mortality in elderly patients over 65 years of age with severe infectious diseases requiring admission to the ICU. In addition, mortality and hospital LOS were significantly elevated in severely ill elderly patients with an ED LOS longer than 24 hours.

In Cardoso's study, delayed admission to the ICU in patients with severe illness led to a higher mortality rate than immediate admission, and each additional hour spent waiting was independently associated with a 1.5% increase in the risk of ICU mortality and a 1.0% increase in the risk of in-hospital mortality [10]. However, this previous study was not conducted with elderly individuals aged 65 years or older but with all patients admitted from the ED to the ICU for severe illness. These patients had various diseases associated with relatively higher mortality rates, such as cardiac arrest, stroke, and myocardial infarction. In Chalfin's study, delayed transfer (>6 hours) from the ED to the ICU was associated with prolonged hospital LOS and mortality [11]. However, in this study, there was no significant difference in in-hospital LOS or all-cause mortality when an ED LOS cutoff value of 6 hours was used. Elderly patients with ED LOS >24 hours had a prolonged hospital LOS (20 vs 15 days) and a higher all-cause in-hospital mortality rate (41.9% vs 27.2%). The present study focused on elderly patients, which means that the target population differed from those in previous studies. The difference in ED LOS cutoff could be due to the effect of regional ED overcrowding with a lack of available ICU beds and elevated bed occupancy rate [17]. ED overcrowding is known to be associated with ED LOS and adverse outcomes in patients visiting the ED for respiratory disease and chest pain [18–20].

It is known that patients aged 85 years or older have a longer ED LOS than younger patients [21]. It is highly likely that the number of elderly individuals visiting the ED will continue to increase in the future and that the ED LOS of elderly individuals may also increase. Elderly patients with infectious diseases visiting the ED often have preexisting diseases and may progress to sepsis, resulting in high rates of mortality and severe illness. Therefore, ED LOS may have a greater effect on elderly patients than younger patients. In our study of elderly patients with critical infectious illnesses, a longer ED LOS was an independent risk factor for all-cause in-hospital mortality, with an odds ratio of 1.01, similar to the results of previous studies. In one study of patients who visited the ED, a longer ED LOS was associated with higher in-hospital cardiac arrest and mortality rates, which is also consistent with the findings of this study. Other study has also suggested that age (OR: 1.02), cancer (1.26), and ED LOS (OR: 1.10) were independent factors associated with the occurrence of cardiac arrest in hospitals [22].

The prevalence of malignancies increases with increasing age. The proportion of severely ill elderly patients who visit the ED with comorbid malignancies is higher than that in other age groups [23]. In addition, patients with malignancies often have immunosuppression due to chemotherapy or the disease itself, thus making it more likely that they will progress to sepsis, shock, and death [23, 24]. In this study, comorbid malignancies were associated with higher all-cause in-hospital mortality, with the highest odds ratio for mortality of 3.76 found in critically ill elderly patients with infections.

Serum lactate is a useful biomarker that can be assessed in patients with infectious diseases who are suspected of having sepsis in the ED. In several diseases, such as sepsis, shock, ischemic stroke, and pulmonary embolism, elevated lactate levels have been shown to be related to mortality [25–30]. In our study, septic shock was identified in 44.7% of the nonsurvivors, and the level of lactate, as an independent factor related to mortality, was significantly different between survivors and nonsurvivors.

The recently updated SSC recommends rapid antibiotic administration within 1 hour after the recognition of the condition for sepsis and septic shock [31]. Previous studies have shown a positive association between delays in antibiotic use and sepsis-related mortality [32, 33]. Nevertheless, another meta-analysis reported results of no difference in mortality between immediate (0 to 1 hour after onset) and early (1 to 3 hours after onset) antibiotic administration in patients with severe sepsis or septic shock [34]. Additionally, the present study did not show a difference in door-to-antibiotic time between nonsurvivors and survivors. The different results may be due to differences in the characteristics of the target group. In future studies, assessment of antibiotic administration time and outcome may be needed to better elucidate risk factors for sepsis in the elderly.

One limitation of this multicenter study on elderly patients over 65 years of age with infectious diseases was the absence of comparisons with noninfectious diseases and other age groups. This was a retrospective study, which was subject to selection bias due to missing and incomplete data. Additionally, elderly individuals who died within 24 hours of visiting the ED were not included in this study, so there may have been selection bias. This study considered factors that could affect mortality, such as comorbidities, initial KTAS level, and initial sepsis-related biomarkers in the ED, but there were still unmeasured confounders of the Charlson comorbidity index. In addition, each ED participating in this study recommended the same sepsis management in accordance with the recently updated SSC guidelines but did not evaluate the association between the volume of fluid resuscitation and in-hospital mortality of elderly patients due to the limitations of retrospective studies. In addition, a long-term prospective study is needed because the sample size was relatively small.

In conclusion, this study shows that prolonged ED LOS is independently associated with all-cause in-hospital mortality in elderly patients with infections requiring ICU admission. An ED LOS longer than 12 hours was associated with an increased hospital LOS in severely ill elderly patients. ED LOS should be considered in the development of strategies to prevent adverse outcomes in elderly patients with infections visiting the ED.

## Figures and Tables

**Table 1:** Classification of infections in critically ill elderly patients.

Classification	All (*n* = 439)	Nonsurvivor (*n* = 132)	Survivor (*n* = 307)	*p* value
Pneumonia	271 (61.7)	89 (67.4)	182 (59.3)	0.108
Genitourinary infection	69 (15.7)	16 (12.1)	53 (17.3)	0.175
Hepatobiliary infection	47 (10.7)	9 (6.8)	38 (12.4)	0.084
Gastrointestinal infection	21 (4.8)	9 (6.8)	12 (3.9)	0.190
Others	31 (7.1)	9 (6.8)	22 (7.2)	0.896

**Table 2:** Baseline characteristics of critically ill elderly patients with infection.

Characteristic	All (*n* = 439)	Nonsurvivor (*n* = 132)	Survivor (*n* = 307)	*p* value
Age (years) (IQR)	78 (73–83)	79 (73–82.5)	78 (73–83)	0.996
Gender, male, *n* (%)	222 (50.6)	71 (53.8)	151 (49.2)	0.376

*Comorbidities, n (%)*				
Diabetes mellitus	176 (40.1)	56 (42.4)	120 (39.1)	0.513
Hypertension	264 (60.1)	81 (61.4)	183 (59.6)	0.731
Chronic renal disease	64 (14.6)	19 (14.4)	45 (14.7)	0.943
Cardiovascular disease	54 (12.3)	17 (12.9)	37 (12.1)	0.809
Malignancy	72 (16.4)	39 (29.5)	33 (10.7)	<0.001

*Initial vital signs*				
Systolic BP (mmHg)	120.5 (98–141)	114 (93.5–136.5)	122 (98–146)	0.036
Diastolic BP (mmHg)	70 (57–80)	66 (54–80)	70 (54.5–80)	0.603
Heart rate (beats/min)	96 (80–112)	96 (80–111)	96 (84–118)	0.578
Respiratory rate (breaths/min)	20 (20–24)	20 (18–22)	20 (20–24)	0.067
Body temperature (°C)	36.8 (36.2–37.9)	36.9 (36.2–37.9)	36.8 (36.3–37.9)	0.545
KTAS level (1, 2), *n* (%)	182 (41.5)	56 (42.4)	126 (40.0)	0.788

*Laboratory results, median (IQR)*				
WBC count (10^9^/L)	11.6 (7.9–18.2)	11.6 (7.6–18.4)	11.5 (7.8–17.0)	0.542
Platelets	207.0 (145.0–291.0)	175.5 (113.0–263.5)	214.0 (156.0–299.5)	0.007
Total bilirubin	0.8 (0.5–1.3)	1.0 (0.6–1.5)	0.8 (0.5–1.2)	0.022
Albumin	3.2 (2.7–3.6)	3.0 (2.6–3.4)	3.2 (2.7–3.6)	<0.001
Lactate	1.9 (1.2–3.4)	2.3 (1.5–4.3)	1.7 (1.2–2.8)	<0.001
SOFA score	6 (4–9)	8 (5–11)	5 (3–8)	<0.001
Septic shock, *n* (%)	123 (28.0)	59 (44.7)	64 (20.8)	<0.001
Door-to-antibiotic time (min)	188 (138–272)	174 (131–261)	194 (143–275)	0.140
Vasopressor at ED, *n* (%)	184 (41.9)	83 (62.9)	101 (32.9)	<0.001
Ventilator at ED, *n* (%)	155 (35.3)	66 (50.0)	89 (29.0)	<0.001
ED LOS (hours)	8.0 (5.0–19.0)	9.0 (5.1–28.4)	8.0 (5.0–16.5)	0.023
ICU LOS (days)	6.0 (3.0–12.0)	7.5 (4.0–14.0)	6.0 (3.0–12.0)	0.050
Hospital LOS (days)	16.0 (9.0–28.0)	13.5 (6.0–24.0)	17.0 (10.0–29.0)	0.002

BP: blood pressure; KTAS: Korean Triage and Acuity Scale; WBC: white blood cell; SOFA: Sequential Organ Failure Assessment; ED: emergency department; LOS: length of stay; ICU: intensive care unit.

**Table 3:** Multivariate logistic regression model of independent risk factors for all-cause in-hospital mortality.

	Univariate analysis			Multivariate analysis		
	Odds ratio	95% CI	*p* value	Odds ratio	95% CI	*p* value
Malignancy	3.48	2.07–5.86	<0.001	3.76	1.88–7.52	<0.001
Systolic BP	0.99	0.99–1.00	0.035	1.00	0.99–1.01	0.971
Platelets	0.99	0.99–1.00	0.147			
Albumin	0.55	0.40–0.76	<0.001	0.76	0.49–1.19	0.235
Lactate	1.20	1.09–1.32	<0.001	1.13	1.01–1.27	0.039
SOFA score	1.19	1.12–1.26	<0.001	1.06	0.95–1.18	0.279
Septic shock	3.07	1.98–4.77	<0.001	1.24	0.59–2.58	0.575
Vasopressor at ED	3.46	2.26–5.29	<0.001	1.35	0.60–3.06	0.467
Ventilator at ED	2.45	1.61–3.73	<0.001	1.48	0.77–2.83	0.242
ED LOS	1.01	1.01–1.02	<0.001	1.01	1.00–1.02	0.039

BP: blood pressure; ED: emergency department; LOS: length of stay.

**Table 4:** Outcomes according to the ED LOS in critically ill elderly patients with infection.

ED LOS cutoff	Characteristic	<6 h (*n* = 179)	>6 h (*n* = 260)	*p* value
6 hour	ICU LOS	6 (3–12)	7 (3–13)	0.692
	Hospital LOS	16 (8–27.5)	16 (9–29.5)	0.501
	All-cause mortality	50 (27.9)	82 (31.5)	0.418

		<12 h (*n* = 298)	>12 h (*n* = 141)	

12 hour	ICU LOS	6 (3–10)	7 (3–15)	0.121
	Hospital LOS	15 (8–27)	18 (11–35)	0.018
	All-cause mortality	85 (28.5)	47 (33.3)	0.305

		<24 h (*n* = 353)	>24 h (*n* = 86)	

24 hour	ICU LOS	6 (3–11)	8 (3–18)	0.097
	Hospital LOS	15 (9–27)	20 (11–36)	0.044
	All-cause mortality	96 (27.2)	36 (41.9)	0.008

ICU: intensive care unit; ED: emergency department; LOS: length of stay.

## Data Availability

All data used to support the findings of this study are available from the corresponding author upon request.
